# Value of CT in COVID-19-pandemia: A systematic analysis of CT-findings and outcomes in patients with COVID-19 pneumonia

**DOI:** 10.1097/MD.0000000000034359

**Published:** 2023-07-14

**Authors:** Nima Nadem Boueini, Patrick Haage, Nadine Abanador-Kamper, Lars Kamper

**Affiliations:** a Witten/Herdecke University, Witten, Germany; b Clinical Radiology, Helios University Hospital Wuppertal, Wuppertal, Germany; c Department of Cardiology, HELIOS Medical Center Wuppertal, Germany.

**Keywords:** chest CT, COVID 19, prognostic assessment, outcome, ICU, RSNA report template

## Abstract

Chest-computer tomography (CT) is a crucial factor in the clinical course and evaluation of patients with COVID-pneumonia. In the initial phase of the COVID-19 pandemic little information was known on the prognostic value of the initially taken thoracic CTs. The purpose of this study was to determine predictive values for clinical outcome based on CT classification of the pulmonary pathologies in patients with COVID-pneumonia. This single center study included 51 non-immunized patients during the first COVID-19 outbreak in Germany. The patients underwent a clinically indicated chest-CT. Using the radiological society of North America (RSNA)-report template, chest-CTs were classified into 4 categories (typical, atypical, indeterminate, and no changes). We analyzed the outcomes based on these imaging classifications and relevant comorbidities. Among the 51 patients of our study population 14 (27.5%) patients had a lethal outcome. Typical radiological COVID-19 pattern was found in 92.9% of the deceased patients and in 59.5% of the surviving patients (*P* = .022). The lethal group showed a significant higher proportion of diabetes mellitus (50% vs 10.8%; *P* = .003) and arterial hypertension (aHTN) (85.7% vs 54.1%; *P* = .037). Male sex, higher age and coronary heart disease (CHD) were also seen more often in the lethal group. In patients with clinically proven COVID-19 pneumonia, typical chest CT findings show a negative outcome. A classification system used in this study is helpful for classifying imaging features and is recommended as a standardized CT reporting tool. It could also help in triaging of the therapy of patients with COVID-19 pneumonia. Especially the comorbidities, diabetes and arterial hypertonia triggered a negative outcome in our study population.

## 1. Introduction

In 2019, a SARS-COV-2 associated pneumonia was reported in Wuhan, China and rapidly spread worldwide. COVID-19 often leads to a severe pneumonia and was classified as a global pandemic by the WHO in December 2019.^[1]^ Definitive diagnosis was based on real-time reverse transcriptase-polymerase chain reaction (RT-qPCR).^[2]^

Pulmonary computer tomography (CT)-scan is key for the detection of pulmonary manifestations and the prognostic assessment.^[3]^ Thus, the short examination time of the CT-scan compared to that of the PCR-tests is of specific advantage, especially in the emergency setting. The CT-scans of patients with COVID-19 display specific pulmonary findings.^[4]^ Aksu et al for example analyzed typical pulmonary CT findings in COVID-19 patients with splenomegaly.^[5]^

The Radiological Society of North America (RSNA) released a consensus document on the reporting chest findings related to COVID-19.^[6]^ It categorizes chest-CT findings, which alleviates the decision of whether the possibility of a COVID-19-pneumonia is given or not.

We aimed to analyze the influence of the 4 different RSNA categories on the prognosis of patients with COVID-19 pneumonia. Therefore, main aspect of the study was the examination of specific chest CT patterns and their impact on the patients outcome.

## 2. Material and methods

### 2.1. Study design and patients

**Figure 1.**
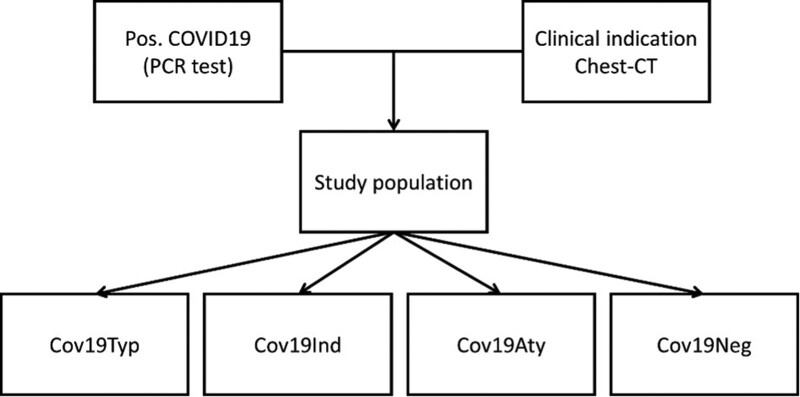
Schematic flow chart of the study population.

The study is performed as a retrospective study; patients with COVID 19 who were admitted to our hospital from March 2020 to August 2020 were evaluated. Our hospital is a university hospital with roughly 1000 beds and is one of the largest hospitals in the region, therefore, many patients with COVID-19 pneumonia were treated here. Figure 1 shows the flowchart of the study. The ethics-committee (Ethics Committee of Witten-Herdecke University) reviewed the procedure and declared it to be ethical.

### 2.2. Inclusion criteria

The inclusion criteria were a positive PCR-Test and a clinically indicated CT of the patients due to their critical condition.

### 2.3. Chest-CT-protocols

All images were obtained using a Somatom Definition Flash (Siemens Medical Systems, Germany) in the supine position during end-inspiration. The main scanning parameters were as follows: voltage: 100 KV, kernel B30f and B70f, collimation: 0.6 to 5 mm. Tube current: 368 to 760 mAs.

### 2.4. Image analysis

The CT-Scans that were analyzed by experienced radiologists from our radiological department. The RSNA reporting scheme was introduced at the early beginning to standardize the reporting of COVID-19-CTs. The CT-scans were classified using the RSNA-scheme in typical, atypical, negative and indeterminate (Table 1).

**Table 1:** RSNA-reporting template modified from Simpson S, Kay FU, Abbara S, et al Radiological Society of North America Expert Consensus Statement on Reporting Chest CT Findings Related to COVID-19. J Thorac Imaging. 2020 Jul;35 (4):219–227.

COVID-19 classification	CT-findings
Typical appearance	•Peripheral, bilateral GCO with or without consolidation or visible intralobular lines (“crazy-paving”) •Multifocal GCO of rounded morphology with or without consolidation or visible intralobular lines •Reverse halo sign or other findings of organizing pneumonia (seen later in the disease)
Indeterminate appearance	•Absence of typical features AND Presence of: •Multifocal diffuse perihilar or unilateral GCO with or without consolidation lacking a specific distribution and are nonrounded and non-periphereal. •Few very small GCO with a nonrounded and non-periphereal distribution
Atypical appearance	•Absence of intermediate and typical features AND Presence of: •Isolated lobar or segmental consolidation without GCO. •Discrete small nodules (centrilobular, “tree in bud”) •Lung cavitation. •Smooth interlobular septal thickening with pleural effusion.
Negative for pneumonia	•No CT features to suggest pneumonia

CT = computer tomography, RSNA = radiological society of North America.

### 2.5. Baseline characteristics

Baseline characteristics as age, sex and comorbidities as arterial hypertonia, coronary heart disease (CHD), chronic obstructive pulmonary disease (COPD), asthma, diabetes mellitus, peripheral artery disease, cardiac arrhythmia and obesity were included in the analysis to examine their potential prognostic influence. It can be assumed that those chosen parameters could have a significant effect on the outcome in patients with COVID-19 pneumonia, like other infectious respiratory diseases.

### 2.6. RSNA-template used for structural reporting

The report template (Table 1) shows 4 categories as seen in the table above: the typical ([Cov19typ|), the indeterminate ([Cov19ind]), and the atypical ([Cov19aty]). The fourth category indicated that there were no signs of pneumonia.

### 2.7. Statistical analysis

Statistical analyses were performed using IBM SPSS Statistics Software (IBM, Chicago, IL). We categorized the patient population based on the outcome into 2 groups: surviving patients (NL-group, “non-lethal”) versus lethal outcome (L-group, “lethal”). The comparisons were evaluated using the *t* test, Mann to Whitney *U* test or Chi square test. A *P* value of <.05 was defined as statistically significant.

## 3. Results

### 3.1. Baseline characteristics

We analyzed 51 patients, 37 of the patients survived their COVID-19 Infection (37/51, 72.5%), 14 patients died (14/51, 27.5%). Of the study population, 31 patients were male and 20 were women. In the NL-group 22 patients were male (22/37, 59.5%) and 15 persons were female (15/37, 40.5%), the L-group consisted of 9 (9/14, 64.3%) male and 5 female patients (5/14, 35.7%).

The mean age in the NL-group was 64 years, while that in the L Group was 76 years.

Out of the 37 in the NL-group 20 people had arterial hypertension (aHTN) (20/37, 54.1%). In the L-group, 12 patients had aHTN (12/14, 85.7%). Seven of the 37 (7/37, 18.9%) in the NL-group and six of the 14 patients (6/14, 42.9%) in the L-group had CHD. Five patients in the NL-group (5/37, 13.5%) and 2 in the L-group (2/14, 14.3%) had COPD.

In the L-group 2 people had asthma (2/14, 14.3%), in the NL-group 4 patients had asthma (4/37, 10.8%).

Four patients in the L-group had obesity type I (4/14, 28.6%) and 2 patients were overweight (2/14, 14.3%). In the NL-group 10 patients were overweight, 7 patients had obesity type I, 2 patients had obesity type II and 1 person had obesity type III.

Four patients in the NL-group and 7 persons in the L-group had type II diabetes.

Table 2 summarizes the baseline characteristics.

**Table 2:** Baseline characteristics for NL-group and L-group.

Baseline	Non-lethal (NL) n = 37		Lethal (L) n = 14		
	n	%/MV ± SD	n	%/MV ± SD	*P* value
Sex					
Male	22	59.5%	9	64.3%	
Female	15	40.5%	5	35.7%	
Age (yr)		64 ± 3		76 ± 3	
aHTN	20	54.1%	12	85.7%	.037
CHD	7	18.9%	6	42.9%	.08
COPD	5	13.5%	2	14.3%	.944
Asthma	4	10.8%	2	14.3%	.734
DM	4	10.8%	7	50%	.003
PAD	1	2.7%	0	0%	.538
Cardiac arrhythmia	3	8.1%	3	21.4%	.192
Obesity:					
Normal	17	45.9%	8	57.1%	.48
Overweight	10	27.0%	4	28.6%	.91
Adipositas I	7	18.9%	2	14.3%	.87
Adipositas II	2	5.4%	0	0%	.38
Adipositas III	1	2.7%	0	0%	.54

For categorical variables, non-parametric Chi-squared (χ^2^) test or Fisher exact test were applied. For all tests, a common *P* value < .05* was determined statistically significant.

aHTN = arterial hypertension, CHD = coronary heart disease, COPD = chronic obstructive pulmonary disease, DM = diabetes mellitus, MV = mean value, PAD = peripheral artery disease, SD = standard deviation.

### 3.2. Imaging findings

We found typical changes (COVID-[typ]) in the chest-CT in 13 of 14 persons (13/14; 92.9%) of the L-group, compared to 22 patients in the NL-group (22/37; 59.5%) (Table 3).

**Table 3:** Results of image analysis: Imaging findings according to RSNA report template.

	Non-lethal		Lethal		*P* value (pearson-chi-square)
	n = 37		n = 14		
	*n*	*%*	*n*	*%*	
COVID-[typ]	22	59.5	13	92.9	.022
COVID-[ind]	7	18.9	0	0	.08
COVID-[aty]	2	5.4	1	7.1	.8
COVID-[0]	6	16.2	0	0	.1

RSNA = radiological society of North America

Indeterminate changes (COVID-[ind]) were documented in 7 patients (7/37, 18.9%) in the NL-group and in none patients in the L-group.

We documented atypical findings (COVID-[aty]) in 2 patients with COVID-infection (2/37, 5.4%) and 1 patient in the L-group (1/14, 7.1%).

Normal pulmonary CT findings (COVID-[0]) were observed in 6 patients of the NL-group (6/37; 16.2) and in none of the patients in the L-group.

All patients in the L-group showed a bilateral pulmonary involvement and 12 patients in the showed an involvement of all 5 lobes of the lung (12/14, 85.7%). In the NL-group 28 patients (28/37, 75.7%) showed a bilateral involvement and 3 (3/37, 8.1%) patients showed unilateral infiltrations of the lung. The specific lobular pattern of the COVID-pneumonia is given in Table 4.

**Table 4:** Results of image analysis: Further morphological findings.

	Non-lethal n = 37		Lethal n = 14		
	*n*	*%/MV ± SD*	*n*	*%/MV ± SD*	*P* value
0 Lobe	6	16.2%	0	0%	.20
1 Lobe	2	5.4%	0	0%	.38
2 Lobes	1	2.7%	0	0%	.28
3 Lobes	3	8.1%	2	14.3%	.30
4 Lobes	2	5.4%	0	0%	.38
5 Lobes	23	62.2%	12	85.7%	.27
Bilateral	28	75.7%	14	100%	.083
Unilateral	3	8.1%	0	0%	.380
Mediastinal Lymphadenopathy	12	32.4%	9	64.3%	.041
Pl. effusion	9	24.3%	6	42.9%	.2
Intubation	2	5.4%	4	28.6%	.023
ECMO	2	5.4%	1	7.1%	.816

ECMO = extracorporeal membrane oxygenation.

Mediastinal lymphadenopathy was seen in 9 patients in the L-group (9/14, 64.3%) and in 12 patients in the NL-group (12/37, 32.4%).

Pleural effusion was detected in 6 patients in the L-group (6/37, 42.9%) and 9 patients in the NL-group (9/37, 24.3%).

Four patients in the L-group (4/14, 28.6%) were intubated, whereas in the NL-group 2 patients (2/37, 5.4%) were intubated.

## 4. Discussion

### 4.1. Demographic findings

This study revealed a slightly higher mortality for male patients with COVID-19 compared to female patients (64% vs 36%). This is consistent with previous findings of higher mortality and a more severe COVID-19 pneumonia in male patients.^[7]^

We observed a higher age of the patients in the L-group than in the NL- group (76 vs 64 years). This is in line with previous publications where patient´s age was a risk factor for a poorer outcome in patients with COVID-19 pneumonia.^[8]^ In addition, the rate of comorbidities was higher in patients with advanced age.

Approximately 85% of the patients who died had aHTN. In the NL-group, about 54% had aHTN. An explanation for this observation is that similar to the age-factor, people who suffer from aHTN are usually older. However, there could be a connection between aHTN and a higher risk of death in cases of COVID-19 pneumonia.

About 43% of L-group had a CHD, whereas only 19% of the NL-group had CHD. This means that less than the half of the patients who died had CHD. However, the fact that many people have an undiagnosed CHD should be considered.

Only 14% in the NL-group and L-group had COPD. This is interesting, because one would expect poorer outcomes in patients with COPD. Another study described a slightly increased mortality in patients with COPD; however, in an age-standardized comparison, this difference was not observed.^[9]^ Due to the small number of patients with COPD, the specific categories (GOLD I-IV) were not examined further.

Similar to COPD, only a small number of patients had asthma as a comorbidity. Only 14% of the L-group and about 10% of the NL-group had asthma.

50% of the patients in the L-group had diabetes. In the NL-group, only 11% had diabetes. This result was highly significant in terms of the lethal outcome of patients with COVID 19 (*P* = .003). Other studies have also shown that the presence of diabetes significantly increases severe and lethal outcome.^[10]^ Interestingly, a more recent study also showed higher rates of diabetes in patients who had COVID 19- Infection.^[11]^ There are researches analyzing a correlation between oxygen saturation, diabetes, heart rate and COVID-19 using noninvasive systems, which could bring more efficient data in the future.^[12]^

Obesity has been reported as a risk factor for a more severe outcomes in patients with COVID-19 pneumonia.^[13]^ Remarkably, our data do not support this, with no severe adipose patients (type 2 or 3) in the L-group.

### 4.2. Imaging findings

**Figure 2.**
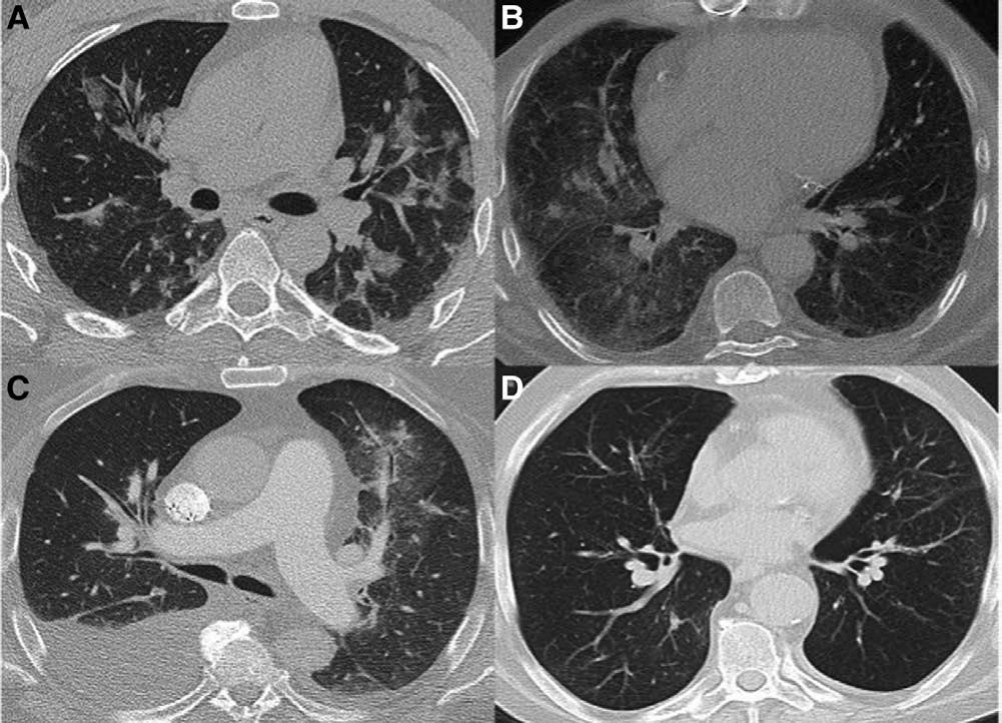
Typical changes: Peripheral, bilateral multifocal ground glass opacities (A). Indeterminate changes: Unilateral diffuse ground glass opacity (B). Atypical changes: Segmental nodular changes (“tree in bud”) and right sided pleural effusion (C). No changes despite clinically proven COVID-pneumonia (D).

The main aim of this study was to analyze imaging findings from COVID-19 CT-scans and to detect prognostic features for the outcome of COVID-19 pneumonia. The RSNA-report template allows an easy categorization of the pulmonary CT-scans. Figure 2 shows examples of the pulmonary patterns on the CT-scans. Concerning the imaging results patients with a poor outcome had mostly (93%, *P* value: .02) typical pulmonary changes. Only one patient showed an atypical pattern. In the L-group, none of the patients showed an indeterminate COVID-19 or a normal pulmonary pattern. This underlines the poor prognosis of pulmonary manifestation of COVID-19.

In the NL-group, 22 patients showed a typical pattern (22/37, 59.5%), 7 had indetermined and 2 patients showed atypical pulmonary changes. Normal pulmonary CT without signs of pneumonia was found in 6 patients in the NL-group.

Typical chest-CT findings of patients with COVID-19-pneumonia are peripheral bilateral GCOs with or without consolidation or with interlobular thickening (“crazy-paving”). In advanced stages of the disease, a reverse-halo sign can be observed. Mediastinal lymphadenopathy and pleural effusion were atypical.^[14]^ In the later stages of the disease, some patients develop changes like fibrosis and/or lung scarring.

Patients who were intubated during the CT-scan had a worse outcome than those who were not intubated (*P* = .02).

In an accuracy analysis of COVID-19 categorization systems, Van Berkel et al proved that those systems such as Covid reporting and data system and CT involvement score^[15]^ are valuable tools for the evaluation of pulmonary COVID-19 infections in a high-prevalence areas. This supports the validity of employing the RSNA template in the prognostic assessment of COVID infections.

### 4.3. Prognostic assessment

So far, still only few data are available on the prognostic value of specific characterization tools, such as the RSNA-template, and specific morphological CT-pattern in patients with COVID-19 pneumonia.

In line with our data, previous studies have demonstrated the overall prognostic value of Chest-CT depending on pulmonary involvement.^[16]^ In addition, our study included the first prognostic assessment using the RSNA-template.

Another study analyzed the influence of lung-involvement and laboratory parameters on the patients’ outcomes. The authors reported a proportional relationship between lung involvement and patient´s mortality and the prognostic superiority of CT-findings compared to nonspecific inflammation markers.^[17]^ This is consistent with our findings. Our study adds significant clinical risk factors for a lethal outcome (e.g., aHTN and diabetes mellitus). Scharf et al demonstrated a high predictive value for a negative outcome using a combination of pulmonary involvement and laboratory inflammation markers in COVID-19 pneumonia.^[18]^

Aksu et al analyzed the relationship among splenomegaly, Lung Involvement Patterns and Severity Score in COVID-19 pneumonia^[5]^ In their study they showed that Consolidation, interlobular septal thickening, tuberculosis sequela, pleural band, and crazy pavement patterns were frequent in the COVID-19 pneumonia patients with splenomegaly. Our prognostic findings of specific pulmonary pattern may complement these scores. In addition, previous studies analyzed the influence of further factors, like lymphocyte-ratios on pulmonary involvement and prognostic outcome.^[19]^

Our data were able to identify with COVID-19 patients with potential negative outcome based on chest-CT findings. Typical patterns in chest-CT are a prognostic marker for a negative outcome. These findings may be an addition to the algorithm of Ahmed & Alsisi for the identification of patients who need immediate intensive care unit care.^[20]^

### 4.4. Limitations

This study was conducted early during the first pandemic outbreak in Germany between March and August 2020. Thus, this study has some limitations. At the beginning of the pandemic, only a few patients were eligible. This led to a small sample size, especially in the lethal-group (L-group). Despite this fact, our data were able to suggest that typical findings in COVID-CT may predict a poorer outcome than non-typical findings. Since the first pandemic outbreak several mutations of the virus occurred, so there is further investigation needed on how the newly mutated viruses affect the CT-patterns of the lung.

## 5. Conclusion

The RSNA report template allows a safe identification of typical pulmonary patterns in patients with COVID-19 pneumonia. These typical findings were associated with poorer outcomes in our study population especially compared to patients with untypical pulmonary findings. These results could aid the assessment of the disease prognosis as well as clinical triage in patients with COVID-19 pneumonia and potentially adapt the treatment accordingly. Certain comorbidities analyzed in this study, such as diabetes and aHTN were associated with a negative outcome.

The small number of subjects requires confirmation by studies with a larger study population.

Following studies are needed to clarify the relationship between certain comorbidities and specific pulmonary CT-patterns. Further research is necessary to confirm our findings for upcoming virus mutations.

## Author contributions

**Conceptualization:** Nima Nadem Boueini, Patrick Haage, Nadine Abanador-Kamper, Lars Kamper.

**Data curation:** Nima Nadem Boueini, Lars Kamper.

**Formal analysis:** Nima Nadem Boueini, Lars Kamper.

**Investigation:** Nima Nadem Boueini, Lars Kamper.

**Methodology:** Nima Nadem Boueini, Lars Kamper.

**Project administration:** Nima Nadem Boueini, Patrick Haage, Nadine Abanador-Kamper, Lars Kamper.

**Resources:** Nima Nadem Boueini, Lars Kamper.

**Software:** Nima Nadem Boueini, Lars Kamper.

**Supervision:** Patrick Haage, Lars Kamper.

**Validation:** Nima Nadem Boueini, Nadine Abanador-Kamper, Lars Kamper.

**Visualization:** Nima Nadem Boueini, Lars Kamper.

**Writing – original draft:** Nima Nadem Boueini, Lars Kamper.

**Writing – review & editing:** Nima Nadem Boueini, Patrick Haage, Lars Kamper.
